# Supplementary material for: Collaborative intelligence in AI: Evaluating the performance of a council of AIs on the USMLE
Source: PLOS Digit Health. 2025 Oct 9;4(10):e0000787. doi: 10.1371/journal.pdig.0000787 (PMC12510544; doi:10.1371/journal.pdig.0000787)
Supplement: S2 File — (ZIP) [file pdig.0000787.s002.zip › S2 Data Raw data files containing unprocessed question inputs and LLM outputs including transcripts of deliberations/STEP3_Question-137-1_Type-MC-NJ.docx]

**Facilitator** **[Round 1]:** A 31-year-old man with a 5-year history of HIV infection comes to the office because of anal pain, particularly on defecation, for the past 4 months. He says he has seen spots of blood on the toilet tissue but has not had any other noticeable bleeding. He reports no change in bowel habits and has not had recent fever, chills, or rectal drainage. He says he and his partner engage in anal-receptive intercourse. His most recent CD4+ T-lymphocyte count 2 months ago was 350/mm³; HIV viral load at that time was undetectable. He currently is being treated with antiretroviral therapy. He has had no opportunistic infections. Medical history is also significant for syphilis and genital herpes treated with penicillin and acyclovir, respectively. He does not smoke cigarettes or drink alcoholic beverages. Vital signs are normal. Physical examination shows small bilateral inguinal lymph nodes, but respiratory, cardiac, and abdominal examinations disclose no abnormalities. There are several tender fleshy lesions around the perianal area. Rectal examination produces tenderness, but there is no rectal discharge. Test of the stool for occult blood is trace positive. Which of the following is the most appropriate pharmacotherapy at this time? (A) Acyclovir (B) Imiquimod (C) Levofloxacin (D) Metronidazole (E) Penicillin

**AI_B:** RESTATING THE QUESTION: The question pertains to an HIV positive male enduring anal pain particularly during defecation along with intermittent bleeding for the past 4 months. There are fleshy lesions around the perianal area and his stool tested positive for occult blood. Previous health history includes genital herpes and syphilis that were treated. The patient is currently on antiretroviral treatment for HIV and the viral load is undetectable. We are asked to select the most appropriate pharmacotherapy for this patient's current condition. PLANNING: The facts presented show that the patient has fleshy, perianal growths, experiences tenderness during a rectal examination, and tests positive for trace occult blood. These findings, along with the patient's history of anal-receptive intercourse and HIV infection, lead me to consider human papillomavirus (HPV) infection as a strong possibility which manifests typically as anal warts in this clinical setting. Therefore, the first assumption is that the anal pain and bleeding are due to these warts which are likely caused by HPV. CLINICAL REASONING: The absence of systemic symptoms such as fever or chills and local symptoms like rectal discharge supports the idea that this isn't due to systemic or local bacterial infection, ruling out the necessity of antibiotics or antiviral treatment. Considering the patient's known history of syphilis and genital herpes, we can also dismiss penicillin and acyclovir as treatments as they are used for these diseases which have already been treated. CONCLUDING: Based on these clinical presentations and exclusions, the HPV infection assumption seems to be the most likely one standing. This brings our clinical thought process to the question - what is the best treatment for warts caused by HPV infection. RESPONSE: Topical therapies, particularly imiquimod, is a modality used commonly for the treatment of genital and perianal warts and is indeed the most appropriate pharmacotherapy for this patient's clinical situation. Hence, the correct answer would be (B) Imiquimod.

**AI_D:** (1) RESTATING THE QUESTION: A 31-year-old man, under treatment for HIV infection and with a history of syphilis and genital herpes, is experiencing anal pain, particularly during defecation, and has noticed small spots of blood on the toilet paper. There are tender lesions around his anus, he shows a slightly positive result for hidden fecal blood but no other abnormal signs. Given his history and the clinical presentation, we are asked to select the most appropriate drug treatment from the following five options: acyclovir, imiquimod, levofloxacin, metronidazole, and penicillin. (2) PLANNING: Now, we pipe these findings and the options provided through our reasoning process. Anal pain, fleshy lesions, and spots of blood, could mean many things. Considering his history of engaging in anal-receptive intercourse, it increases the possibility of sexually transmitted infections (STIs) with resultant anorectal complications. The major assumption is that he has an anogenital wart infection, which is a common STI. It could also be haemorrhoids, anal fissures, perianal abscess, or ischemic proctitis, but given his sexual habits, a consideration for anal warts should be at the top. (3) CLINICAL REASONING: Anal warts are caused by the Human Papilloma Virus (HPV). We also need to bear in mind that the CD4+ T-lymphocyte count, an indication of the patient's immune status, is 350/mm³, which is lower than average, indicating immunosuppression. As such, he may be more susceptible to infections such as HPV. The anal pain and bleeding are likely due to these warts becoming irritated and possibly ulcerated during defecation. Imiquimod is an immune response modifier used to treat external genital and perianal warts caused by HPV. (4) CONCLUDING: Given the clinical findings in the question stroke, the history of receptive anal intercourse, and the current symptoms, the most likely diagnosis is anal warts caused by HPV. The recommended treatment in such scenarios, from the options given, is topically applied Imiquimod. (5) RESPONSE: Therefore, the most appropriate pharmacotherapy for the patient at this time is Imiquimod.

**AI_C:** RESTATING THE QUESTION: A patient who is HIV-positive, treated with antiretroviral therapy, and has a medical history of syphilis and genital herpes for which he was treated, presents with symptoms of anal pain and bleeding specifically during defecation. He also noticed tender perianal lesions. Upon inspection, several tender, fleshy lesions were found around the perianal area. The question wants to know the best course of pharmacotherapy for this patient. PLANNING: The presence of tender, fleshy perianal lesions along with the given symptoms requires an evaluation of the possible conditions that could lead to such manifestations. The presence of tender, fleshy lesions raises suspicion of HPV (Human Papillomavirus) infection. Various treatments can effectively manage HPV infections, including acyclovir, imiquimod, levofloxacin, metronidazole, and penicillin. However, to choose the most appropriate treatment, we first need to identify the type of HPV infection. Given the patient's symptoms and the appearance of the lesions, HPV causing anogenital warts is likely. CLINICAL REASONING: Anogenital warts are benign epithelial tumors that are typically caused by low-risk HPV types 6 and 11. These warts can occur in the perianal area, which aligns with our patient's presentation. Imiquimod, an immune response modifier used for the treatment of external genital and perianal warts, could be a hypothetical solution. However, reconsidering all the conditions and treatments, only Imiquimod is directly indicated for anogenital warts caused by HPV. CONCLUDING: Judging the patient's symptoms, along with physical examination and medical history, the most likely diagnosis is HPV causing anogenital warts. These warts typically appear as soft, moist, or flesh-colored swellings in the genital and anal areas, which our patient is experiencing. Consequently, considering the effectiveness of different medications in managing such warts, imiquimod appears to be the most appropriate pharmacotherapy at this point. RESPONSE: Hence, based on the symptoms and medical condition of the patient, we should opt for 'Imiquimod' (option B) at this time.

**AI_A:** (1) RESTATING THE QUESTION: Our HIV-positive patient, who has a history of syphilis and genital herpes as well but doesn't smoke or drink, is currently experiencing anal pain with defecation, evidenced by spot bleeding but not noticeable blood. He has not had any fever, chills, or rectal discharge, and his bowel habits haven't noticeably changed either. His latest CD4+ count a couple of months back was reasonable and his HIV is well under control with antiretroviral therapy. However, the physical examination has found several tender and fleshy lesions around his perianal area and these are most likely the source of his discomfort. A rectal examination provoked tenderness but no discharge, and occult blood testing of his stool has revealed trace amounts. The question is asking for the most suitable pharmacotherapy for our patient's condition. (2) PLANNING: Considering his sexual activity, there is a relatively high chance that his symptoms could be due to a sexually transmitted disease (STD). The possibilities here are several: condyloma acuminatum (genital warts) caused by HPV, syphilis, herpes, or a bacterial infection such as gonorrhea or chlamydia. Each of these STDs has a specific treatment: (A) Acyclovir is used for genital herpes, (B) Imiquimod for genital warts, (C) Levofloxacin for bacterial infections like gonorrhea and chlamydia, (D) Metronidazole for bacterial vaginosis and trichomoniasis, and (E) Penicillin for syphilis. (3) CLINICAL REASONING: Our patient recovering from syphilis and herpes means Acyclovir and Penicillin were previously effective, and syphilis and herpes can presumably be ruled out due to lack of associated classic symptoms. However, fleshy, tender lesions around the anal area, which this patient exhibits, are classic manifestations of HPV infection, a virus known for causing condyloma acuminatum (genital warts). Given his sexual behavior, our patient is at significant risk of HPV infection. This would lend credibility to the assumption that he has an HPV infection expressing as condyloma acuminatum. (4) CONCLUDING: Taking into account the patient's sexual habits, the symptoms present, and the physical examination findings, it is clinically reasonable to hypothesize that the patient is suffering from condyloma acuminatum, caused by HPV - a condition known for causing painful, fleshy growths around the anal area. (5) RESPONSE: Therefore, based on our train of thought, the most suitable pharmacotherapy at this point for our patient ought to be (B) Imiquimod, which is used for the treatment of genital warts caused by HPV.

**AI_E:** Restating the question: A 31-year-old HIV-positive male has presented with anal pain especially upon defecation, spots of bloody tissue, no noticeable changes in bowel habits, and absence of febrile symptoms or rectal discharge for the past 4 months. His CD4+ T-lymphocyte count was 350/mm³ two months ago and his HIV viral load was undetectable at that time. He has been compliant with his antiretroviral therapy and has no history of opportunistic infections. Past treatment includes penicillin and acyclovir for syphilis and genital herpes, respectively. He doesn't smoke or consume alcohol. Although small inguinal lymph nodes were seen, no respiratory, cardiac, or abdominal anomalies were detected. Tender fleshy perianal lesions were observed, alongside tenderness upon rectal examination, without rectal discharge. Occult blood was traced in the stool sample. The query is to determine the most suitable pharmacotherapy for his case. Planning: An enduring anal pain with spots of blood being noticed is suggestive of anorectal or perianal disorders. The man engages in anal-receptive intercourse, a factor that could expose him to sexually transmitted infections (STIs) such as HPV, which can result in anorectal or perianal abnormalities. Both CD4+ T-lymphocyte count and HIV viral load levels indicate that his HIV is currently well controlled, hence opportunistic infections are less likely here. Yet, tender fleshy lesions around the perianal area and trace amount of blood in stools hint at localized infection, inflammation, or growth. Therefore, the critical assumption to make would be that this patient's symptoms are largely related to an STI that has caused the perianal lesions. Clinical Reasoning: Given the clinical scenario and the fact that the patient is engaged in anal-receptive intercourse, a sexually transmitted infection causing the perianal lesions seems plausible. HPV is prevalent in patients who practice anal-receptive intercourse and often presents as perianal warts, which could be the tender fleshy lesions seen. Symptoms of HPV-induced perianal warts can include anal pain and minor bleeding, both present in this patient. Although HIV patients are susceptible to other STIs and opportunistic infections, his CD4+ T-lymphocyte count and undetectable viral load, in alignment with the lack of fever, systemic symptoms, or rectal discharge, render it less likely that an opportunistic infection or systemic bacterial infection is responsible for his symptoms. Concluding: With clinical reasoning, it can be hypothesized that this man's perianal issues are due to an STI, tentatively perianal warts induced by the HPV virus. The suitable pharmacotherapeutic approach for these types of warts would be a treatment with imiquimod, a topical immunomodulator that spurs local immune response against the virus. Response: (B) Imiquimod is the most appropriate pharmacotherapy for this patient at this time. It can be applied topically to stimulate a local immune response against the HPV virus, thus treating the perianal warts and associated symptoms.

**Facilitator** The consensus statement synthesizes the responses of AI_A, AI_B, AI_C, AI_D, and AI_E as follows: The question pertains to a 31-year-old HIV-positive man who has been experiencing anal pain, particularly during defecation, and has noticed spots of blood on the toilet paper for the past four months. He has a history of syphilis and genital herpes, both of which have been treated, and his HIV is well-controlled with antiretroviral therapy. Physical examination reveals tender, fleshy lesions around the perianal area, and a stool test shows trace amounts of occult blood. Given these symptoms and his history, the question asks for the most appropriate pharmacotherapy. The patient's symptoms, sexual behavior, and physical examination findings suggest a high likelihood of a sexually transmitted disease (STD), specifically an HPV infection manifesting as condyloma acuminatum (genital warts). The tender, fleshy lesions around the anal area are classic manifestations of this condition. The absence of systemic symptoms such as fever or chills and local symptoms like rectal discharge supports the idea that this isn't due to systemic or local bacterial infection, ruling out the necessity of antibiotics or antiviral treatment. Given the patient's known history of syphilis and genital herpes, we can also dismiss penicillin and acyclovir as treatments as they are used for these diseases which have already been treated. Therefore, the most suitable pharmacotherapy for this patient, considering his symptoms and the effectiveness of different medications in managing such warts, is Imiquimod. This topical immunomodulator is commonly used for the treatment of genital and perianal warts caused by HPV. In conclusion, based on the patient's symptoms, sexual habits, and medical history, the consensus among the AI responses is that the most appropriate pharmacotherapy for this patient at this time is Imiquimod (option B).
